# Regulation of SOX10 stability via ubiquitination-mediated degradation by Fbxw7α modulates melanoma cell migration

**DOI:** 10.18632/oncotarget.5639

**Published:** 2015-10-09

**Authors:** Xiao-Bin Lv, Wei Wu, Xiaofeng Tang, Yanqing Wu, Yinghua Zhu, Yujie Liu, Xiuying Cui, Junjun Chu, Pengnan Hu, Jingjing Li, Qiannan Guo, zeming Cai, Juan Wu, Kaishun Hu, Nengyong Ouyang

**Affiliations:** ^1^ Nanchang Key Laboratory of Cancer Pathogenesis and Translational Research, the Third Affiliated Hospital, Nanchang University, Nanchang, China; ^2^ Guangdong Provincial Key Laboratory of Malignant Tumor Epigenetics and Gene Regulation, Medical Research Center, Sun Yat-Sen Memorial Hospital, Sun Yat-Sen University, Guangzhou, China; ^3^ Department of Obstetrics and Gynecology, Sun Yat-Sen Memorial Hospital, Sun Yat-Sen University, Guangzhou, China; ^4^ Breast Tumor Center, Sun Yat-Sen Memorial Hospital, Sun Yat-Sen University, Guangzhou, China; ^5^ Cancer Center of Guangzhou Medical University, Guangzhou, China

**Keywords:** SOX10, Fbxw7α, ubiquitination, melanoma, GSK3β

## Abstract

Dysregulation of SOX10 was reported to be correlated with the progression of multiple cancer types, including melanocytic tumors and tumors of the nervous system. However, the mechanisms by which SOX10 is dysregulated in these tumors are poorly understood. In this study, we report that SOX10 is a direct substrate of Fbxw7α E3 ubiquitin ligase, a tumor suppressor in multiple cancers. Fbxw7α promotes SOX10 ubiquitination-mediated turnover through CPD domain of SOX10. Besides, GSK3β phosphorylates SOX10 at CPD domain and facilitates Fbxw7α-mediated SOX10 degradation. Moreover, SOX10 protein levels were inversely correlated with Fbxw7α in melanoma cells, and modulation of Fbxw7α levels regulated the expression of SOX10 and its downstream gene MIA. More importantly, SOX10 reversed Fbxw7α-mediated suppression of melanoma cell migration. This study provides evidence that the tumor suppressor Fbxw7α is the E3 ubiquitin ligase responsible for the degradation of SOX10, and suggests that reduced Fbxw7α might contribute to the upregulation of SOX10 in melanoma cells.

## INTRODUCTION

SRY-related HMG box-containing factor 10 (SOX10) is a transcription factor that belongs to the HMG-box transcription factor family; this protein is initially expressed in premigratory neural crest cells and controls the multipotency, survival, and proliferation of neural crest cells as well as their differentiation into peripheral glial cells and pigment cells at later stages [1]. In addition to its role as a multipotency factor in stem cells, SOX10 has been implicated in the expression of lineage-specific genes in glia and melanocytes [2–4]. Homozygous deletion of SOX10 in mice leads to embryonic lethality, whereas SOX10 haploinsufficiency results in a melanocytic phenotype with reduced pigmentation of the belly and limb extremities [5, 6]. Previous studies have revealed low frequencies of intragenic mutations of the Sox10 gene in metastatic melanoma, suggesting that SOX10 might be involved in mediating melanoma metastasis [7]. Upregulation of SOX10 protein has been observed in multiple cancer types, including melanocytic tumors and tumors of the nervous system. More recently, a critical role for SOX10 in tumorigenesis and melanoma migration has been demonstrated in cell lines and mouse models [8–11].

SOX10 expression is tightly regulated at the transcriptional level. Fourteen multiple-species conserved sequences (MCS) were reported to display high levels of evolutionary conservation and variable control of *Sox10* expression [12–14]. SOXE was identified as binding to MSC4 and MSC7 and thereby enhancing the expression of *Sox10*. Moreover, four transcriptional factors were found to directly activate *Sox10* transcription [1, 13]. Autoregulation of *Sox10* has been shown in Schwannoma cells [3]. Recently, *Sox10* expression was shown to be directly activated in immortalized mammary gland epithelial cells by the TRAP/Drip/Mediator complex, which includes Mediator complex subunit 1 (MED1) and activates gene transcription. MED1 is recruited to the *Sox10* promoter at MCS4 and MCS7, and knockdown of MED1 expression completely ablates *Sox10* expression in this cell line [15]. The regulation of SOX10 protein at the posttranslational level is less well understood. One study suggested that sumoylation at K55, K246 and K357 of SOX10 by Ubc9 repressed the transcriptional activity of SOX10 [16]. However, the mechanism by which SOX10 protein stability is regulated remains unknown.

Fbxw7 is the substrate recognition component of the Skp1-Cul1-F-box (SCF) ubiquitin-ligase SCF^Fbxw7^ [17]. Mammals express three alternatively spliced Fbxw7 isoforms (Fbxw7α, Fbxw7β and Fbxw7γ) that are localized in the nucleus, cytoplasm and nucleolus, respectively [17]. Fbxw7 contains an F-box domain of ∼40 amino acids (which interacts directly with Skp1 to recruit ubiquitin-conjugating enzymes) and eight WD40 repeats (which are required for its association with substrates) [18, 19]. Substrates bind to Fbxw7 through a conserved phosphodegron (CPD), ΦxΦΦΦ(T/S)PPx(T/S/E/D), where Φ represents hydrophobic residues, and T/S is phosphoserine or phosphothreonine [17]. Many studies from different groups have identified a growing list of specific Fbxw7 substrates, such as Aurora A, Cyclin E, c-Myc, c-Jun, c-Myb, Hypoxia-inducible factor-1α, Krüppel-like factor 5, Myeloid cell leukemia-1 (Mcl-1), mammalian target of rapamycin, Neurofibromatosis type 1, Notch, Nuclear factor E2-related factor 1, JUNB, Sterol regulatory element-binding proteins, Mediator 13, Krüppel-like factor 2, NF-κB2 and Granulocyte colony stimulating factor receptor (G-CSFR) [20]. Fbxw7 has been characterized as a general tumor suppressor in human cancer, and reduced Fbxw7 expression is often observed in multiple human cancers, including breast cancer, colorectal cancer, gastric cancer, prostate cancer, pancreatic cancer and hepatocellular carcinoma [17]. Moreover, emerging evidence has shown that Fbxw7 controls stem cell self-renewal, cell fate decisions, survival, and multipotency in numerous tissues, including the hematopoietic [21] and nervous systems [22, 23], liver [24, 25], adipose tissue [26], endothelium [27], intestine [28], lung [29] and pancreas [30]. Because of the important role of Fbxw7 in various physiological and pathological processes, novel Fbxw7 substrates and biological functions of Fbxw7-mediated protein turnover are of great interest.

In this study, we revealed that SOX10 is an unstable protein, and its stability is controlled by the ubiquitin-proteasome proteolytic pathway. Further studies identified Fbxw7α as a potential E3 ubiquitin ligase responsible for SOX10 turnover. Fbxw7α bound to and facilitated the ubiquitination-mediated degradation of SOX10 through phosphodegron. This process is promoted by glycogen synthase kinase 3β (GSK3β)-mediated phosphorylation of SOX10 at the CPD motif. More importantly, we found that Fbxw7α suppresses melanoma cell migration by promoting SOX10 proteolysis. These findings help us to understand the post-translational regulatory mechanism of SOX10 and the underlying clinical significance of the Fbxw7α-SOX10 axis in melanoma.

## RESULTS

### SOX10 is an unstable protein

**Figure 1:**
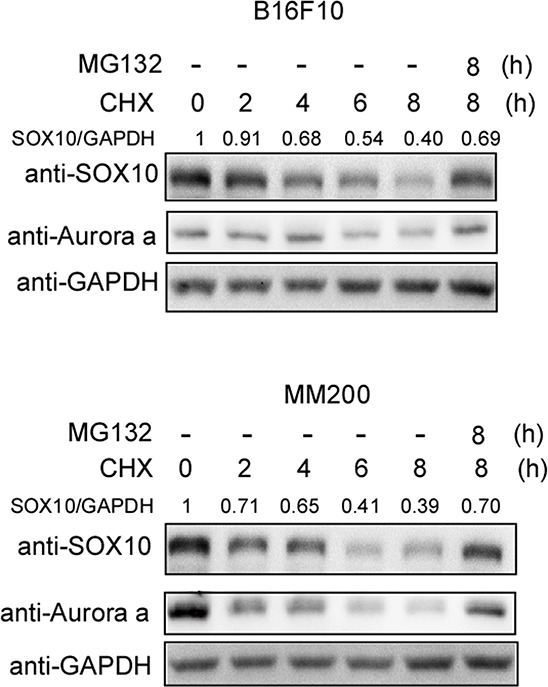
SOX10 is an unstable protein in melanoma cells B16F10 (upper panel) and MM200 (bottom panel) cells were treated with 20 μg/ml cycloheximide (CHX) and 10 μM MG132 for the indicated times before harvest. The cells were then analyzed by Western blotting with the indicated antibodies (*n* = 3).

To determine whether the SOX10 protein is stable, we assessed the half-life of SOX10 in melanoma cells using the cycloheximide (CHX) chase assay. Aurora-a, a validated unstable protein [31], was used as a positive control. As shown in Figure 1, the SOX10 protein level decreased steadily following protein synthesis inhibition by CHX treatment. The half-life of SOX10 was approximately 4 h. In addition, proteasome inhibitor MG132 treatment induced SOX10 accumulation, suggesting that SOX10 degradation was mediated by ubiquitination.

### SOX10 interacts with Fbxw7α

**Figure 2:**
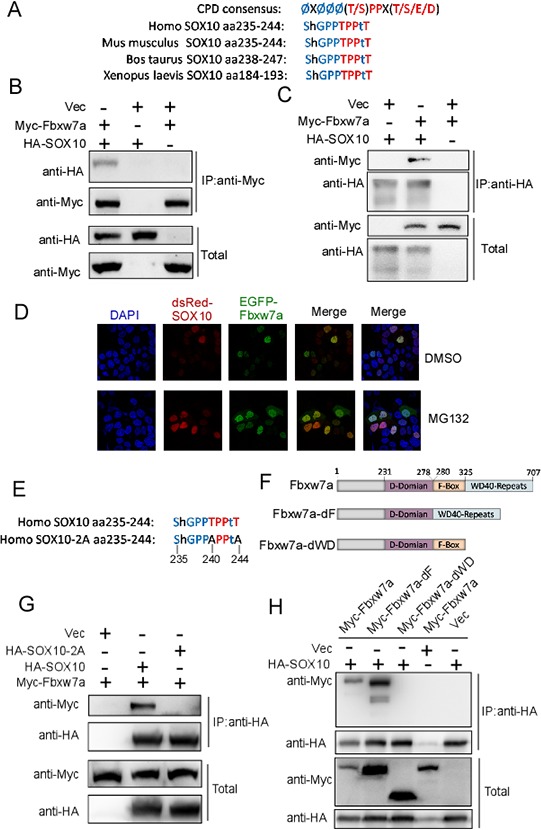
SOX10 is a potential substrate of Fbxw7α **A.** Schematic illustration of the potential CPD sequence in SOX10. **B.** and **C.** SOX10 interacts with SOX10. 293T cells transfected with the indicated plasmids for 24 h were lysed. Immunoprecipitation (IP) using anti-HA (B) or anti-Myc (C) agarose and Western blotting with the indicated antibody were performed (*n* = 3). **D.** Co-localization of Fbxw7α and SOX10 in Hela cells. Hela cells cotransfected with EGFP-Fbxw7α and dsRed-SOX10 for 24 h were fixed and dyed with DAPI. Then the Co-localization of Fbxw7α and SOX10 was observed in the confocal microscope. **E, F.** Schematic illustration of the SOX10–2A mutant (E) and Fbxw7α truncation (F). **G.** Mutation of the SOX10 CPD sequence abolished the interaction of SOX10 with Fbxw7α. 293T cells transfected with the indicated plasmids for 24 h, and the formation of an immunoprecipitated complex was detected as described in Figure 2B (*n* = 3). **H.** Fbxw7α interacts with SOX10 and this interaction is dependent on the WD40 domain. 293T cells were transfected with the indicated plasmids for 24 h, and the formation of an immunoprecipitated complex was detected as described in Figure 2B (*n* = 3).

To explore the molecular mechanisms of SOX10 degradation, we sought to identify the E3 ubiquitin ligase responsible for this degradation. Analysis of the amino acid sequence of SOX10 revealed a potential conserved CPD identified in numerous Fbxw7 substrates located between amino acids 235 and 244 of SOX10 (Figure 2A). Considering that SOX10 is a transcription factor [32] which is usually localized in nucleus, we examined the possibility that SOX10 is a potential substrate of Fbxw7α, the only Fbxw7 isoform localizing in nucleus. Firstly, we tested whether SOX10 interacted with Fbxw7α using co-immunoprecipitation (co-IP). HA-tagged SOX10 was co-transfected with or without Myc-tagged Fbxw7α into 293T cells, and reciprocal co-IP using anti-Myc or anti-HA was performed. As shown in Figure 2B and 2C, the complex containing these two proteins was obviously detected in the cell lysates. Furthermore, the co-localization of Fbxw7α and SOX10 was examined by co-transfected of EGFP-Fbxw7a and dsRed-SOX10 into Hela cells. As shown Figure 2D, Fbxw7α and SOX10 were co-localized in nucleus, and MG132 treatment increased their level in nucleus. We next investigated the crucial domains responsible for their interaction. Mutation of the CPD motif (Figure 2E) abrogated the interaction between SOX10 and Fbxw7α (Figure 2G), indicating that the CPD motif was essential for the recognition of SOX10 by Fbxw7α. In addition, deletion of the WD40 domain but not F-box (Figure 2F) abrogated the interaction between Fbxw7α and SOX10 (Figure 2H), indicating that Fbxw7α binds to SOX10 through its WD40 domain.

### Fbxw7α targets SOX10 for ubiquitination

**Figure 3:**
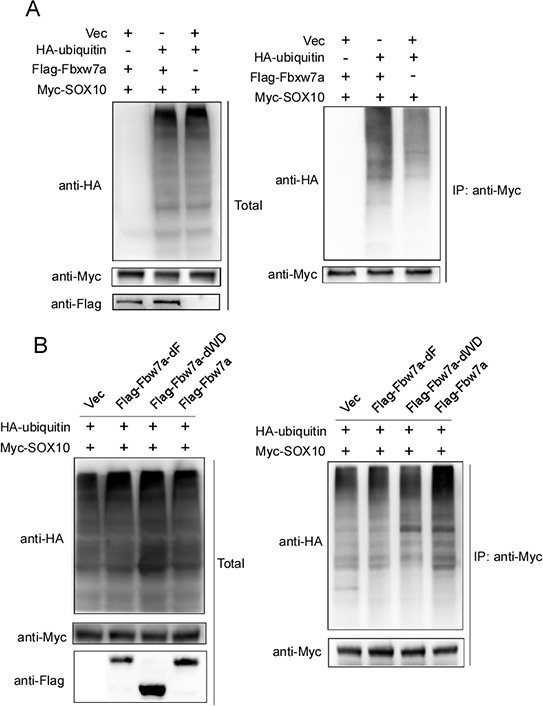
Fbxw7α mediated SOX10 ubiquitination **A.** 293T cells transfected with the indicated plasmids for 24 h were treated with 10 μM MG-132 for 4 h before lysis. Immunoprecipitation (IP) using anti-Myc agarose and Western blotting with the indicated antibody were performed (*n* = 3). **B.** Deletion of either the F-box or the WD40 domain abrogates the ability of Fbxw7α to mediate the ubiquitination of SOX10. 293T cells transfected with the indicated plasmids were treated with 10 μM MG-132 for 6 h before lysis. Analysis of the ubiquitination level of SOX10 was performed as described in Figure 3A (*n* = 3).

Fbxw7α is a component of E3 ubiquitin ligase that promotes the degradation of target proteins through ubiquitination. Thus, we used an *in vivo* ubiquitination assay to test whether Fbxw7α promotes SOX10 ubiquitination. 293T cells transfected with Myc-SOX10 and HA-ubiquitin in the absence or presence of Flag-Fbxw7α were treated with MG132 for 6 h to stabilize the ubiquitinated proteins before lysis. In the absence of ectopic Fbxw7α, SOX10 was weakly ubiquitinated, whereas cotransfection of Fbxw7α increased the ubiquitinated SOX10 level (Figure 3A). Moreover, deletion of either the F-box or WD40 domain abolished Fbxw7α-induced SOX10 ubiquitination (Figure 3B). These results indicate that Fbxw7α facilitates the ubiquitination of SOX10.

### Fbxw7α facilitates the degradation of SOX10

**Figure 4:**
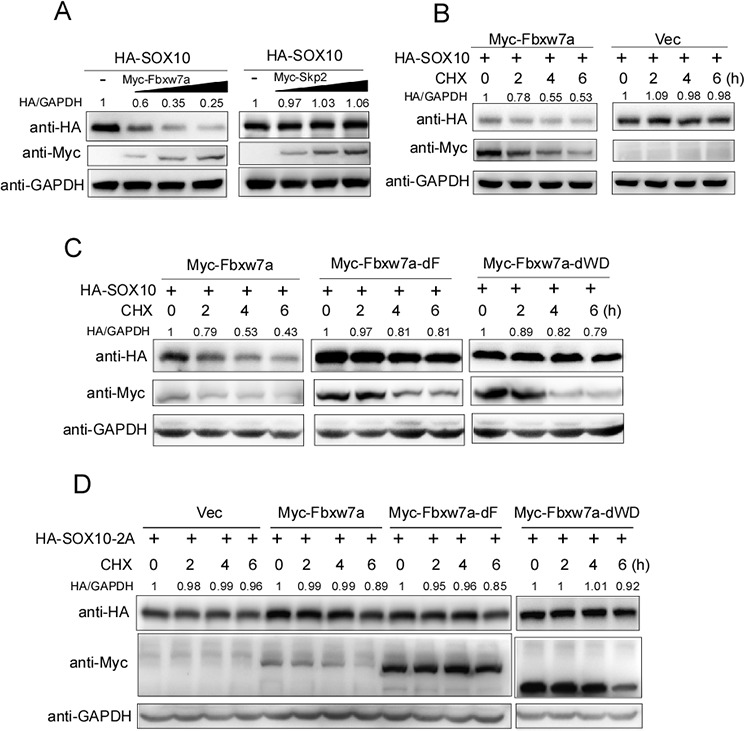
Fbxw7α mediated SOX10 degradation **A.** Fbxw7α transfection downregulates the SOX10 protein level in a dose-dependent manner. 293T cells were transiently transfected with HA-SOX10 and an increasing amount of Myc-Fbxw7α or Myc-Skp2 for 24 h and were examined by Western blotting with the indicated antibodies (*n* = 3). **B.** Fbxw7α transfection accelerates the degradation of SOX10. 293T cells transfected with the indicated plasmids for 24 h were treated with 20 μg/ml CHX for the indicated times. The cells were then harvested and analyzed by Western blotting with the indicated antibodies (*n* = 3). **C.** Deletion of either F-box or WD40 domains abrogates the ability of Fbxw7α to mediate the degradation of SOX10. 293T cells transfected with the indicated plasmids for 24 h were treated with 20 μg/ml CHX for the indicated times and were analyzed as described in Figure 4B (*n* = 3). **D.** Mutation of the CPD leads to the resistance of SOX10 to Fbxw7α-mediated degradation. 293T cells transfected with the indicated plasmids for 24 h were treated with 20 μg/ml CHX for the indicated times and analyzed as described in Figure 4B (*n* = 3).

Based on the observation that Fbxw7α targets SOX10 for ubiquitination, we detected whether Fbxw7α promoted SOX10 turnover. HA-SOX10 was co-transfected with different amounts of Myc-Fbxw7α into 293T cells. Skp2, another F-box containing SCF E3 ubiquitin ligase, was used as a control [33]. Indeed, Fbxw7α overexpression reduced SOX10 protein levels in a dose-dependent manner, whereas Myc-Skp2 overexpression did not affect SOX10 protein levels (Figure 4A). Moreover, ectopic expression of HA-Fbxw7α notably reduced the half-life of SOX10 using the CHX chase assay (Figure 4B), whereas deletion of either the F-box or the WD40 domain abolished Fbxw7α-mediated SOX10 turnover (Figure 4C). Moreover, mutation of the CPD sequence of SOX10 (SOX10–2A) abrogated its degradation by Fbxw7α (Figure 4D). Taken together, these results indicate that Fbxw7α is the E3 ubiquitination ligase that mediates SOX10 degradation.

### GSK3β is required for the Fbxw7α-mediated degradation of SOX10

**Figure 5:**
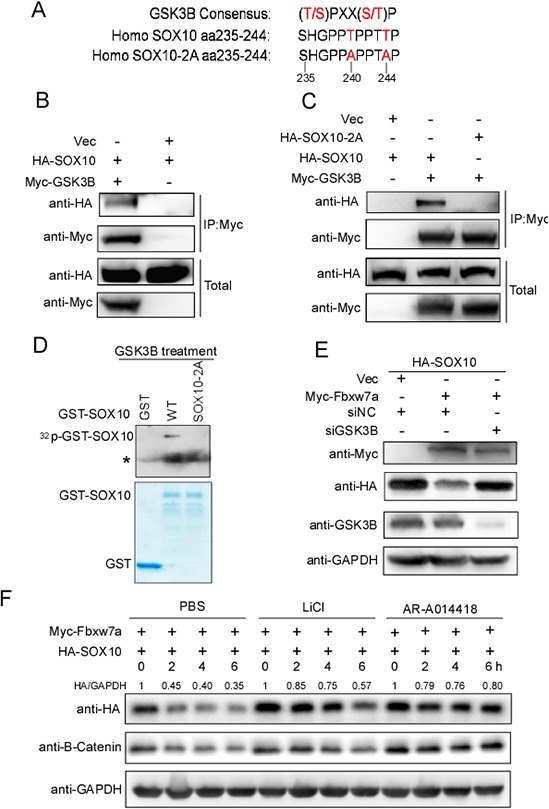
GSK3β is required for Fbxw7α-mediated SOX10 turnover **A.** Schematic illustration of a GSK3β recognition consensus in the SOX10 CPD sequence. **B.** GSK3β interacts with SOX10. 293T cells transfected with the indicated plasmids for 24 h were lysed. Immunoprecipitation using anti-Myc agarose and Western blotting with the indicated antibody were performed (*n* = 3). **C.** Mutation of the GSK3β recognition consensus abrogates the association of SOX10 with GSK3β. 293T cells were transfected with the indicated plasmids for 24 h, and the formation of an immunoprecipitated complex was detected as described in Figure 5B (*n* = 3). **D.** GSK3β phosphorylates SOX10 *in vitro* at CPD domain. GSK3β protein immunoprecipitated from Myc-GSK3β transfected 293T cells was incubated with 5 μg of the indicate GST or GST-SOX10 proteins in the presence of γ-32P-ATP. The kinase reaction products were resolved by SDS-PAGE and phosphorylation was detected by autoradiography (*n* = 3, * indicates non-specific band). **E.** Silencing of GSK3β abrogates, in part, Fbxw7α-mediated degradation of SOX10. 293T cells were transfected with the indicated plasmids, siRNAs were lysed, and the indicated protein levels were examined by Western blotting (*n* = 3). **F.** Treatment with the GSK3β inhibitor LiCl or AR-A014418 abolishes, in part, the Fbxw7α-mediated degradation of SOX10. 293T cells transfected with the indicated plasmids were treated with PBS, 20 mM LiCl or 20 μM AR-A014418 for 24 h. The cells were then treated with 20 μg/ml CHX for the indicated times before harvesting (*n* = 3).

Phosphorylation of T/S in the CPD motif of Fbxw7α substrates is required for recognition by Fbxw7α [34]. We sought to determine which phosphokinase is responsible for the phosphorylation of the SOX10 CPD motif. Scansite software analysis revealed that SOX10 CPD is a potential GSK3β phosphorylation motif (Figure 5A). We next examined the interaction between GSK3β and SOX10 by co-IP. HA-SOX10 was co-transfected with or without Myc-GSK3β into 293T cells, and SOX10 was immunoprecipitated using the anti-HA antibody. GSK3β was copurified with SOX10 only when they were cotransfected (Figure 5B). Furthermore, mutation of SOX10 CPD (SOX10–2A) abrogated their interaction, indicating that GSK3β interacts specifically with SOX10 and that this interaction depends on the potential GSK3β phosphorylation motif in CPD (Figure 5C). More importantly, in a *in vitro* kinase assay, we found that GSK3β directed phosphorylated SOX10, whereas mutation of SOX10 CPD (SOX10–2A) impaired this phosphorylation (Figure 5D).

We further tested whether GSK3β influenced SOX10 expression. HA-SOX10 was co-transfected with Myc-Fbxw7α into 293T cells with or without GSK3β knockdown, and the SOX10 protein level was monitored by Western blotting. The SOX10 protein level was downregulated upon co-transfecting with Fbxw7α, whereas silencing of GSK3β led to elevation of SOX10 levels compared with the siRNA control (Figure 5E). In addition, treatment with the GSK3β inhibitors LiCl or AR-A014418 reversed, at least in part, the Fbxw7α-mediated degradation of SOX10 and increased the half-life of SOX10 (Figure 5F). The half-life of β-Catenin, a well-known GSK3β substrate [35], was also increased upon LiCl or AR-A014418 treatment, indicated that these inhibitors worked well in the conditions(Figure 5F). Taken together, these results indicate that GSK3β could be the phosphokinase for the phosphorylation of the SOX10 CPD motif, and its kinase activity was required for Fbxw7α-mediated degradation of SOX10.

### Fbxw7α regulates endogenous SOX10 in melanoma cells

**Figure 6:**
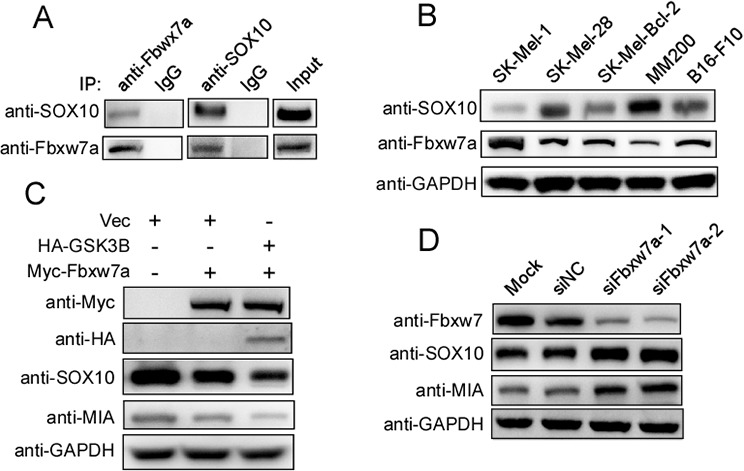
Fbxw7α regulates the SOX10/MIA signaling pathway **A.** Endogenous interaction between SOX10 and Fbxw7α was detected in melanoma cells. MM200 cells were lysed and subjected to IP using IgG, anti-Fbxw7α, or anti-SOX10, as indicated, and then, the cells were analyzed by Western blotting (*n* = 3). **B.** The SOX10 protein level was inversely correlated with Fbxw7α. Various melanoma cell lines as indicated were lysed, and the protein levels of SOX10, Fbxw7α and GAPDH were evaluated by Western blotting. **C.** Overexpression of Fbxw7α and GSK3β downregulates the SOX10 and MIA protein levels (*n* = 3). MM200 cells were lysed, and the indicated protein levels were detected by Western blotting (*n* = 3). **D.** Silencing of Fbxw7α upregulates the SOX10 and MIA protein levels. SK-Mel-Bcl2 cells were lysed, and the indicated protein levels were detected by Western blotting (*n* = 3).

To further investigate the regulatory relationship between Fbxw7α and SOX10, we first examined the endogenous interaction between Fbxw7α and SOX10 in melanoma cells. A co-IP assay showed that the complex containing the two proteins was obviously detected in melanoma cells using either anti-Fbxw7α or anti-SOX10 antibodies (Figure 6A). We next examined whether Fbxw7α regulated the endogenous SOX10 level in melanoma cells. Fbxw7α and SOX10 protein levels were detected by Western blotting in a panel of melanoma cells. As shown in Figure 6B, SOX10 was inversely correlated with the Fbxw7α protein levels in melanoma cells. Moreover, Fbxw7α overexpression in MM200 cells downregulated SOX10 expression (Figure 6C). MIA was reported to be a transcriptional target of SOX10 and responsible for SOX10 mediated melanoma migration [10]. We hence examined whether Fbxw7α regulated MIA level. Indeed, MIA was also downregulated upon Fbxw7α overexpression. Furthermore, co-transfection of GSK3β with Fbxw7α further reduced the protein level of SOX10 compared with Fbxw7α transfected alone (Figure 6C). By contrast, silencing of Fbxw7α in SK-Mel-Bcl2 cells increased the SOX10 and MIA protein levels (Figure 6D). Taken together, these results indicate that Fbxw7α regulates the endogenous expression of SOX10 in melanoma cells.

### Fbxw7α suppresses melanoma migration through mediation of SOX10 turnover

**Figure 7:**
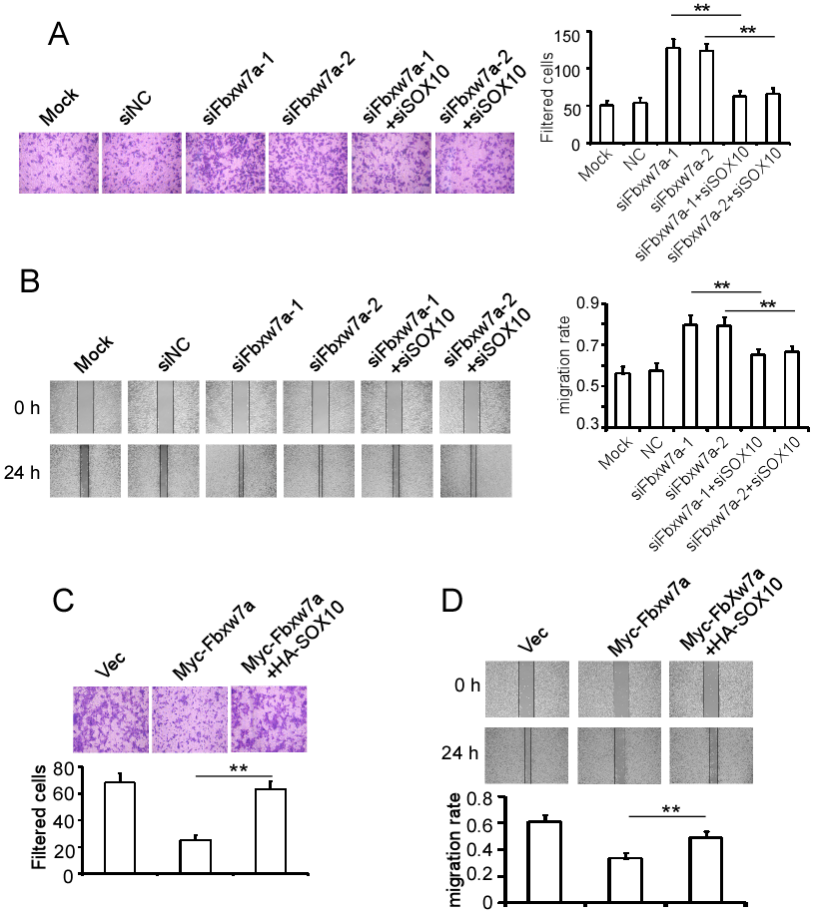
Fbxw7α suppresses cell migration through targeting SOX10 for degradation **A.** and **B.** SK-Mel-Bcl2 cells were transfected with the indicated siRNAs for 48 h, and their migratory ability was tested using a Transwell assay (A) and wound-healing assay (B). **C.** and **D.** MM200 cells were transfected with the indicated plasmids for 24 h, and their migratory ability was tested using the Transwell assay (C) and wound-healing assay (D). The results are expressed as the mean ± SD; *n* = 3, **p < 0.05.

It has been reported that Fbxw7α suppresses the migration of melanoma cells [36]. We investigated the role of SOX10 in Fbxw7α-mediated migratory inhibition of melanoma cells. SK-Mel-Bcl2 cells were transfected with Fbxw7α siRNAs with or without SOX10 siRNA and were subjected to Transwell and wound-healing assays. The Transwell assay showed that Fbxw7α siRNA transfection dramatically increased the filtered SK-Mel-Bcl2 cells, whereas co-transfection of SOX10 siRNA reduced the filtered cells similar to the negative control (Figure 7A). Consistently, the wound-healing assay showed that co-transfection of SOX10 siRNA reversed the elevation of the migratory ability of SK-Mel-Bcl2 cells induced by Fbxw7α silencing (Figure 7B). By contrast, ectopic expression of Fbxw7α suppressed the migration of MM200 cells, whereas the combined transfection of SOX10 reversed Fbxw7α-exerted migration suppression effect using the Transwell assay (Figure 7C) and wound-healing assay (Figure 7D).

## DISCUSSION

In this study, we provide evidence that SOX10 is a direct substrate of SCF^Fbxw7α^. Fbxw7α interacts with and promotes the ubiquitination-mediated degradation of SOX10 depending on its CPD motif. Moreover, the ubiquitination-dependent degradation of SOX10 by Fbxw7α was enhanced by GSK-3β. Furthermore, Fbxw7α-mediated degradation of SOX10 is pathologically relevant, given that SOX10 can reverse the Fbxw7α-mediated migration-suppression effect on melanoma cells.

*Sox10* expression is initiated in neural crest cells as they dissociate from the neural tube, and its expression is maintained during neural crest cell migration. Expression continues in the glial and melanocyte lineages, but *Sox10* is turned off in many other neural crest cell derivatives [5, 32, 37]. Both the mRNA and protein level of SOX10 show restricted patterns of tissue-specific expression, suggesting that they undergo dominant regulation at the transcriptional level under physiological conditions. To explore whether SOX10 level was regulated posttranslational, we first accessed the stability of SOX10 using CHX chase assay. Our results indicate that SOX10 was an unstable protein. Besides, the observation that MG132 restored the SOX10 level suggested SOX10 might be degraded through a ubiquitination dependent manner. We hence search for the potential E3 ubiquitin ligase responsible for SOX10 turnover and identified Fbxw7α was the E3 ligase mediated SOX10 degradation. Our results showed that overexpression of Fbxw7α accelerated the SOX10 protein turnover using CHX chase assay. Interestingly, the Fbxw7α protein levels also decrease with CHX treatment. This observation was consistent with previous report that Fbxw7α was unstable due to autoubiquitylation [38]. SOX10 was found to be overexpressed in many cancers, including melanoma, schwannoma, neurofibroma, salivary gland tumors, astrocytoma and glioma [39]. The role of SOX10 in melanoma metastasis has been reported by several studies. Graf et al. examined a panel of melanoma cells and found that SOX10 mRNA amounts varied among melanoma cell lines and did not correlate with progression stage, whereas its protein level was associated with a more invasive or metastatic phenotype, indicating that the SOX10 protein level is regulated posttranslationally [10]. In this study, we found that Fbxw7α promoted SOX10 degradation in melanoma cells. Moreover, the SOX10 protein level was inversely correlated with Fbxw7α in a panel of melanoma cells. These results suggest a major role of the posttranslational regulation of SOX10 by Fbxw7α in melanoma progression.

Fbxw7α has been found to be involved in numerous cellular processes, including cell proliferation, apoptosis, cell cycle and differentiation [20]. Importantly, Fbxw7α is considered a tumor suppressor protein primarily because Fbxw7α targets multiple well-known oncoproteins, including Cyclin E, c-Myc, c-Jun, Mcl-1, and Notch-1 for ubiquitination-mediated destruction [34]. A recent report suggested that Fbxw7α inhibits melanoma migration and may serve as a prognostic marker. The authors found that both Fbw7 protein and mRNA expression was significantly reduced in nine melanoma cell lines compared with normal melanocytes. Moreover, silencing of Fbxw7α results in a remarkable increase in cell migration and stress fiber formation of melanoma cells. However, the authors observed only a subtle change in Fbxw7α substrates such as Myc and Cyclin E upon modulation of Fbxw7α expression Fbxw7αin melanoma cells. These findings suggest that other Fbxw7α substrates mediate the migration of melanoma cells [36]. In the present study, we determined that SOX10 is a novel target of Fbxw7α. Furthermore, a rescue experiment indicates that SOX10 could reverse Fbxw7α-exerted migration inhibition in melanoma cells. These results indicate that Fbxw7α suppresses melanoma metastasis through targeting SOX10 degradation.

Fbxw7α was reported to be frequently mutated in multiple tumors from the endometrium (15%), large intestine (9%), thyroid (8%), hematopoietic and lymphoid tissue (8%), pancreas (3%), and others [40]. Mutation of Fbxw7α in melanoma was also documented to be inactivated by somatic gene mutation (∼8.1%, *n* = 8) in metastatic melanomas by exome sequencing [41]. The inactivation of Fbxw7α may lead to the accumulation of SOX10, which promotes melanoma migration.

In summary, we have shown that SOX10 protein stability was regulated by Fbxw7α-mediated ubiquitination degradation. We also show that Fbxw7α suppressed the SOX10-mediated migration-promoting effect on melanoma cells. Given the frequent downregulation or inactivation of Fbxw7α in melanoma, these findings may help us further understand the roles of the Fbxw7α-SOX10 axis in melanoma progression. Furthermore, the differentiation and development of melanocytes and glia may prove to be another useful model in understanding Fbxw7α-mediated degradation of SOX10, as the SOX10 protein level is attenuated in the differentiation and development of melanocytes and glia.

## MATERIALS AND METHODS

### Cell lines

Cells (293T, Hela, MM200, SK-Mel-Bcl2, SK-Mel-1, SK-Mel-28, and B16-F10) were cultured in Dulbecco's Eagle's medium (Life Technologies, USA) supplemented with 10% fetal bovine serum (Biological Industries, Israel), 1 mM glutamine, and 100 units/ml each of penicillin and streptomycin.

### Plasmids

Myc tag, HA tag or Flag tag expression cassettes were inserted into pcDNA3.1 to obtain the Myc-pcDNA3.1, HA-pcDNA3.1 and Flag-pcDNA3.1 vectors, respectively. Myc-Fbxw7α and Flag-Fbxw7α were obtained by inserting Fbxw7α cDNA into the Myc-pcDNA3.1 or Flag-pcDNA3.1 vectors. SOX10 cDNA was cloned into the Myc-pcDNA3.1, HA-pcDNA3.1 and pGEX4T-2 plasmids to obtain Myc-SOX10, HA-SOX10 and GST-SOX10 recombinant vectors. HA-Ub was obtained by cloning ubiquitin cDNA into the HA-pcDNA3.1 plasmid. HA-GSK3β and Myc-GSK3β [42] was a kind gift from Dr. Kang (Sun Yat-Sen University Cancer Center, Guangzhou China). Mutations were introduced using the Quik Change site-directed mutagenesis kit (Stratagene, USA), and all mutations were verified by DNA sequencing.

### Antibodies

The human anti-Fbxw7α antibody was obtained from Abcam (ab12292). Human anti-SOX10 (sc-17342) anti-GSK3β (sc-53931) and anti-GAPDH (sc-166574) antibodies were obtained from Santa Cruz Biotechnology. Human anti-MIA (melanoma inhibitory activity) was purchased from Abnova (PAB27627) antibodies. Anti-HA (#3724), anti-Flag (#14793) and anti-Myc (#2278) were from Cell Signaling Technology. Bound primary antibodies were detected with either horseradish peroxidase-conjugated anti-mouse IgG HRP (SA00001–1, Proteintech, China) or horseradish peroxidase-conjugated anti-rabbit HRP (SA00001–2, Proteintech, China), and proteins were visualized by chemiluminescence.

### Transfection experiments

Transfection was performed as described previously [43]. Briefly, cells seeded at 2.5 × 10^5^ cells per well in a 6-well plate or at 1 × 10^6^ cells per 10-cm plate were transfected with 2 μg or 12 μg of plasmid DNA, respectively, using Lipofectamine™ 2000 (Life Technologies, USA).

### RNA interference

Fbxw7α siRNAs were purchased from Qiagen (siFbxw7α-1, Qiagen SI03089240) and Abnova (siFbxw7α-2, H00055294-R01). SOX10 siRNAs were purchased from Dharmacon (Smart pool, L-017192). GSK3β was designed according to previously validated oligonucleotides [42] and synthesized by GenePharma (Shanghai, China). Transfection was performed according to the manufacturer's instructions using Lipofectamine™ RNAiMAX transfection reagent (Life Technologies, USA) and 100 nM siRNA. The transfected cells were incubated at 37°C for 48 h in complete medium and were harvested at the indicated time points.

### Western blotting and immunoprecipitation

Western blotting and immunoprecipitation were performed as described previously [44]. Briefly, cells were lysed in RIPA buffer [50 mM Tris-HCl at pH 8.0, 2 mM DTT, 5 mM EDTA, 0.5% Nonidet P-40, 100 mM NaCl, 1 mM microcystin, 1 mM sodium orthovanadate, 2 mM phenylmethanesulfonyl fluoride (PMSF), 1 × protease & phosphatase inhibitor cocktail (Thermo Scientific, #1861281)], and clarified lysates were resolved by SDS-PAGE and transferred to PVDF membranes for Western blotting using ECL detection reagents (Advansta, USA; R-03025-D25). Alternatively, clarified supernatants were first incubated with anti-Myc-agarose (Santa Cruz, SC-40AC), anti-FLAG-agarose (Sigma, A2220), or anti-HA-agarose (Sigma, A2095) for 2 h to overnight at 4°C, and the precipitates were washed four times with RIPA buffer. To investigate the interaction between SOX10 and Fbxw7α at the endogenous level, the clarified supernatants were first incubated with anti-Fbxw7α or anti-SOX10 for 2 h at 4°C. Protein A/G-agarose was then added and incubated for 2 h to overnight. Precipitates were washed four times with RIPA buffer and analyzed by Western blotting.

### Wound-healing and transwell assays

These procedures were performed as described previously with small modification [45]. Briefly, cells were plated into 6-well-plates and cultured in complete medium supplemented with 20 μM mitomycin C for 24 h. The scraped, acellular area was created with a 200-μL pipette tip. Then the cells were washed with PBS and cultured in DMEM medium with 0.5% FBS and 20 μM mitomycin C. The spread of wound closure was observed after 24 h and imaged under a microscope. Migration assays were performed in modified Boyden chambers with 8-μm pore filter inserts in 24-well plates (BD Transduction, USA). Briefly, 1 × 10^5^ cells suspended in serum-free DMEM were added to the upper chamber of the insert in each well of a 24-well culture plate. FBS was added to the lower chamber as a chemoattractant at a final concentration of 10%. After 8 h, the nonmigrated cells were gently removed with a cotton swab. The migrated cells in the lower part of the chamber were stained with crystal violet, air dried, and imaged.

### *In vitro* kinase assay

GST-SOX10-WT, GST-SOX10–2A were produced in bacteria and purified and eluted as described previously [46]. GSK3β protein was enriched from Myc-GSK3β transfected 293T cells through immunoprecipitation using anti-Myc agarose. Kinase reactions were carried out in a reaction buffer consisting of 50 mM Tris-HCl, pH 7.4, 1 mM DTT, 10 mM MgCl_2_, 5 μCi [γ-32P] ATP, 500 μM ATP, 2 μg of soluble GST-SOX10 and Myc-GSK3β enriched agarose. Reactions were incubated at 30°C for 1 h, then resolved by SDS-PAGE and detected by autoradiography.

### Statistical analyses

Statistical analyses were performed using SPSS 16.0 software (SPSS Inc.). The values were expressed as the mean ± standard deviation (SD) of three independent experiments, and the significance of differences between two groups was calculated using two-tailed Student's *t*-test. *P*-values less than 0.05 were considered significant.
